# Vitamin D and focal brain atrophy in PD with non-dementia: a VBM study

**DOI:** 10.3389/fnhum.2024.1474148

**Published:** 2024-11-29

**Authors:** Yingying Xu, Erlei Wang, Qilin Zhang, Jing Liu, Weifeng Luo

**Affiliations:** ^1^Department of Neurology and Clinical Research Center of Neurological Disease, The Second Affiliated Hospital of Soochow University, Suzhou, Jiangsu, China; ^2^Department of Radiology, The Second Affiliated Hospital of Soochow University, Suzhou, Jiangsu, China

**Keywords:** Parkinson’s disease, vitamin D, cognition, voxel-based morphometry, gray matter volume

## Abstract

**Background:**

The status of vitamin D has been proposed to have an impact on cognition. Gray matter volume (GMV) is a potential marker of cognitive function. We investigated whether lower serum 25-hydroxyvitamin D level was associated with reduced cerebral GMV in Parkinson’s disease with non-dementia (PDND) patients.

**Methods:**

Baseline neuropsychiatric performance and serum 25-hydroxyvitamin D levels were examined in 24 PDND patients and 24 healthy controls (HCs). A set of cognitive scales were used to evaluate the cognition. Voxel-based morphometry (VBM) was performed to calculate each PDND patient’s GMV, based on structural magnetic resonance imaging data. Associations between serum 25-hydroxyvitamin D levels, cognition, and GMV were evaluated.

**Results:**

The serum 25-hydroxyvitamin D levels of the PDND group were significantly lower than those of the HC group. The simple linear regression analyses between serum 25-hydroxyvitamin D levels and the scores of subtests that analyzed cognitive function showed that serum 25-hydroxyvitamin D levels were negatively correlated with Trail Making Test-A scores and positively correlated with Symbol Digit Modalities Test and Auditory Verbal Learning Test scores. Multiple regression analyses revealed a positive correlation between the right fusiform gyrus GMV and serum 25-hydroxyvitamin D levels.

**Conclusion:**

We hypothesized that the lower serum 25-hydroxyvitamin D level in patients with PDND might affect auditory word learning and spatial cognition ability by reducing the gray matter volume of the right fusiform gyrus, thereby leading to deterioration of semantic understanding and memory function.

## 1 Introduction

Parkinson’s disease (PD) is a common neurodegenerative disease in elderly individuals. An estimated 6.1 million people had a PD diagnosis in 2016 worldwide, 2.4 times higher than that in 1990 (Collaborators GBDPsD, 2018). Cognitive impairment, as one of the most important non-motor symptom of PD, affects quality of life, caregiver burden, and health-related costs (Goldman and Sieg, 2020). However, the etiology and pathogenesis of PD are not well understood. Recently, Newmark and Newmark (2007) found that vitamin D deficiency might be associated with PD pathogenesis. Vitamin D has been regarded as a neurosteroid that regulates immunomodulation and brain development and function in adulthood (Bivona et al., 2019). Many researchers have found that multiple parts of the brain, including hippocampus, cortex, expressed both vitamin D receptor and 1α-hydroxylase (Liu et al., 2005). Plenty of evidences have shown that vitamin D can be actively synthesized by neurons and microglia. Furthermore, vitamin D status has been suggested to influence neurocognition (Butler et al., 2011; Dursun and Gezen-Ak, 2019; Zhang et al., 2019).

Voxel-based morphometry (VBM) is a fully automated, whole-brain measurement technique that maps the statistical probability of differences in regional tissue volumes or densities between groups (Yu et al., 2008). Gray matter volume (GMV) is a marker of brain atrophy and a potential marker of cognitive function, based on VBM. GMV has been used to analyze differences in brain structure among different groups, in many diseases, including PD (Ashburner and Friston, 2000; Uchida et al., 2019).

Karakis et al. (2016) showed that low 25-hydroxyvitamin D levels were associated with smaller hippocampal volumes and worse neuropsychological functions in a large community-based sample. In our previous study, serum vitamin D levels in PD patients were significantly lower than those in controls. Further, vitamin D was found to have a protective effect on the cognitive functions of PD with non-dementia (PDND) patients (Karakis et al., 2016). Previous studies mostly focused on the relationship between vitamin D and brain volume in Alzheimer disease (AD) and schizophrenia patients (Lee et al., 2021; Shivakumar et al., 2015). The identification in PD patients has received little attention. Therefore, our objective was to investigate the prospective correlations between serum vitamin D levels, cerebral GMV, and cognitive functions in PDND patients.

## 2 Materials and methods

### 2.1 Participants

Eligible patients were recruited between January 2016 and March 2018 from the neurology clinic and the inpatient department of the Second Affiliated Hospital of Soochow University. All PDND patients were diagnosed according to the United Kingdom Parkinson’s Disease Society Brain Bank clinical diagnostic criteria and the PDD diagnostic criteria, established by the International Movement Disorders Association, in Emre et al. (2007). The diagnosis of PDD needs to meet the following core features: (1) Diagnosis of Parkinson’s disease according to Queen Square Brain Bank criteria. (2) A dementia syndrome with insidious onset and slow progression, developing within the context of established Parkinson’s disease and diagnosed by history, clinical, and mental examination, defined as: (1) Impairment in more than one cognitive domain; (2) Representing a decline from premorbid level; (3) Deficits severe enough to impair daily life (social, occupational, or personal care), independent of the impairment ascribable to motor or autonomic symptoms (Emre et al., 2007). The patients were divided into PDND group, if they cannot meet the criteria. For comparison, 24 sex-and age-matched healthy volunteers with similar levels of education were recruited as a control group.

The education levels of all participants were at least equivalent to the fourth-grade level which is the minimum level of education that can fulfill the assessment. Patients with other significant neurological diseases that affect cognition were excluded. Patients with abnormal liver and kidney function, thyroid disease, parathyroid disease or vitamin D supplements, were also excluded. This study was approved by the Ethics Committee of the Second Affiliated Hospital of Soochow University (JD-LK-2015-109-09), and the participants (or their guardians) have given their written informed consent.

### 2.2 Clinical assessment

Demographic information and clinical characteristics were collected from all patients, including age at onset, sex, body mass index (BMI), disease duration, medical history, and medications. Levodopa (L-DOPA) equivalent dose (LED) was calculated for each patient (Williams-Gray et al., 2007). Motor manifestations were evaluated using Unified Parkinson’s Disease Rating Scale (UPDRS) part III scores and Hoehn-Yahr staging, in the “off” state. To evaluate the non-motor symptoms of PD, cognition was assessed by the Mini-Mental State Examination (MMSE). The Auditory Verbal Learning Test-Huashan Version (AVLT-H), digit span test (DST), Stroop Color-Word Test (SCWT), Symbol Digit Modalities Test (SDMT), Clock Drawing Test (CDT), Rey-Osterrieth complex figure (ROCF), animal fluency test (AFT, naming as many animals as possible in 60 s), Boston Naming Test (BNT), and the Trail Making Test (TMT), parts A (TMT-A) and B (TMT-B), were also used to evaluate cognitive function. Depression was assessed using the Hamilton Depression Rating Scale 24-Item (HAMD-24).

AVLT-H was employed as a semantic categorization memory test in mainland Chinese populations, which evaluates several aspects of verbal episodic memory through a list of 12 words, including short-term and long-term delayed recall as well as recognition (Huang et al., 2023).

The DST was employed to assess working memory. The participants were asked to repeat series of digits that grew progressively longer. The maximum digit span that the participants could repeat in direct and reverse orders formed the forward and backward scores respectively (Zhong et al., 2021).

The SCWT was employed to assess the ability to suppress cognitive interference as well as the speed and accuracy of information processing. The participant was asked to name the ink color while disregarding the word. We analyzed the time taken to complete the task and the accuracy for items where the ink color is not in accordance with the word meaning (Zhong et al., 2021).

Symbol Digit Modalities Test was used to assess information processing speed, visual perception and attention, working memory ability and hand-eye coordination. The participant was asked to match a series of symbols to corresponding digits in accordance with a symbol-digit pairing illustration. We recorded the number of items that were correctly completed within 90 s (Zhong et al., 2021).

Clock Drawing Test was used to assess visuospatial function, the capacity to utilize symbolic and graphic representation, language, semantic memory, and executive function. The participant was asked to draw a watch face and write the number indicating time in the correct position. Then the participant was asked to draw the minute hand and the hour hand, and the time was asked to point to a specific time. The clock drawing test was scored using a 30-point scale (Dong et al., 2020).

Rey-Osterrieth complex figure was used to assess visuospatial structure and visual memory abilities. The participant was asked to copy the figure within 5 min. This was called ROCF copy and was scored from 0 to 36 points. While copying, they were asked to remember the figure. Then, 30 min after copying, they were asked to recall the figure. This was called ROCF delayed recall (Shuai et al., 2017).

Animal fluency test was used to evaluate language expression ability, semantic memory, and executive function. The participant was asked to name as many animals as possible within 1 min. One point was awarded for each animal named, while no score was given for repeating items (Qian et al., 2021).

Boston Naming Test was used to assess adversarial word retrieval, verbal expression, and visual cognition. The participant was asked to name the subjects of 30 pictures without any time restriction (Qian et al., 2021).

Trail Making Test was employed to detect logical thinking ability, spatial cognition ability, and hand-eye coordination ability. TMT-A task required participants to connect 25 numbers in sequence using lines as rapidly as possible. In contrast to TMT-A, in TMT-B, each digit is surrounded by either a circle or a box. The participant was instructed to connect the digits not only according to the numerical order but also in accordance with the order of the intervals between the circle and the box. The time needed to complete the task was recorded (Zhong et al., 2021).

### 2.3 Vitamin D measurement

Fasting serum levels of 25-hydroxyvitamin D were quantified, using an electrochemiluminescence immunoassay (Roche Cobas 6,000, Tokyo, Japan), according to the manufacturer’s instructions.

### 2.4 MRI data acquisition

All 24 PDND patients were evaluated by magnetic resonance imaging (Achieva, Philips Medical Systems, Best, The Netherlands). MRI scanning was performed with a 3-Tesla MR system. T1-weighted images were collected, using a three-dimensional, magnetization-prepared, rapid-acquisition, gradient-echo sequence. The imaging parameters were as follows: repetition time = 7.1 ms, echo time = 3.5 ms, flip angle = 8°, field of view = 220 × 220 mm, matrix = 352 × 352, slice thickness = 1 mm, 155 continuous slices, and scanning time = 3 min and 19 s. The clinical assessment on cognition, blood sampling, and MRI were performed on the same day.

### 2.5 VBM analysis

Utilizing SPM12 (Wellcome Department of Imaging Neuroscience, London, United Kingdom), MRIs were segmented into gray matter, white matter, and cerebrospinal fluid images through a unified tissue segmentation procedure following image-intensity non-uniformity correction. Subsequently, these segmented gray and white matter images were spatially normalized against a customized template in the standardized anatomic space by employing diffeomorphic anatomical registration through exponentiated lie algebra (DARTEL) (Colloby et al., 2014). The gray and white matter volumes within each voxel were preserved by modulating the images with the Jacobian determinants derived from spatial normalization via DARTEL, and then they were smoothed using an 8-mm full-width at half maximum (FWHM) Gaussian kernel (Abe et al., 2010).

### 2.6 Statistical analysis

Data were analyzed using SPSS software, version 23.0. Continuous variables are presented as the mean ± standard deviation. Comparisons between groups were conducted using independent Student’s *t*-tests or chi-square tests. Simple linear regression analyses were used to analyze the correlations between serum 25-hydroxyvitamin D levels and cognitive function. Multiple regression analyses were performed to explore the associations between 25-hydroxyvitamin D levels and GMV. All analyses were adjusted for intracranial volume, age, sex, education level, UPDRSIII scores, disease duration, BMI, and HAMD scores. All *p*-values were two-tailed, and a significance level of 0.05 was used.

## 3 Results

### 3.1 Clinical and demographic characteristics

The demographic information and clinical features of the 48 participants are presented in Table 1. The serum 25-hydroxyvitamin D levels of the PDND group were significantly lower than those of the HC group.

**Table 1:** Clinical and demographic characteristics of the 48 objectives.

	PDND (24)	HC (24)	*P*-value
Sex, M (%)	12 (50%)	14 (58.33%)	0.562
Age, y	63.1 ± 8.5	65.1 ± 5.9	0.330
Education, y	8.1 ± 3.4	10.1 ± 3.0	0.034^*^
HBP, *n* (%)	8 (33.3%)	7 (29.17%)	0.755
DM, *n* (%)	1 (4.17%)	0	1.000
Smoker, *n* (%)	5 (20.83%)	5 (20.83%)	1.000
Alcohol intake, *n* (%)	3 (12.5%)	2 (8.33%)	1.000
BMI, kg/m^2^	24.1 ± 2.7	—	—
Disease duration, y	4.2 ± 3.5	—	—
LED, mg	373.8 ± 339.5	—	—
H&Y	2.0 ± 0.7	—	—
UPDRS III	25.7 ± 10.3	—	—
MMSE	27.2 ± 2.4	28.8 ± 1.3	0.008^**^
MoCA	22.2 ± 5.4	26.5 ± 2.7	0.001^**^
HAMD-24	8.4 ± 5.7	3.4 ± 3.7	0.002^**^
25-hydroxyvitamin D (nmol/L)	41.8 ± 16.0	50.5 ± 9.3	0.027^*^

HBP, high blood pressure; DM, diabetes mellitus; BMI, body mass index; LED, L-DOPA equivalent dose; H&Y, Hoehn-Yahr scale; UPDRS, Unified Parkinson’s Disease Rating Scale; MMSE, Mini-Mental State Examination; MoCA, Montreal Cognitive Assessment; HAMD, Hamilton Depression Rating Scale. BMI is missing in HC. *P < 0.05; **P < 0.01.

### 3.2 Serum 25-hydroxyvitamin D and cognitive function

**Figure 1:**
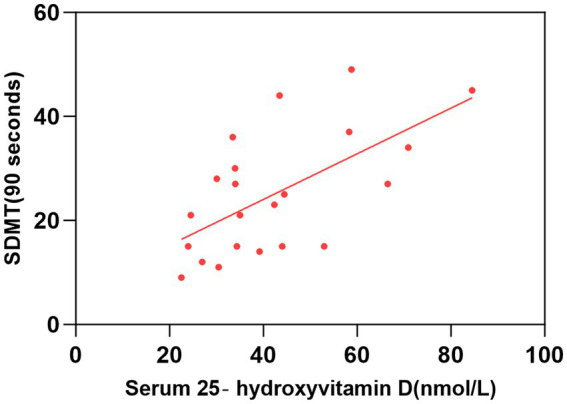
Correlation between serum 25-hydroxyvitamin D level and SDMT (90 s). The simple linear regression analyse showed that serum 25-hydroxyvitamin D levels were positively correlated with SDMT (90 s) scores (*P* < 0.05). SDMT, Symbol Digit Modalities Test.

**Figure 2:**
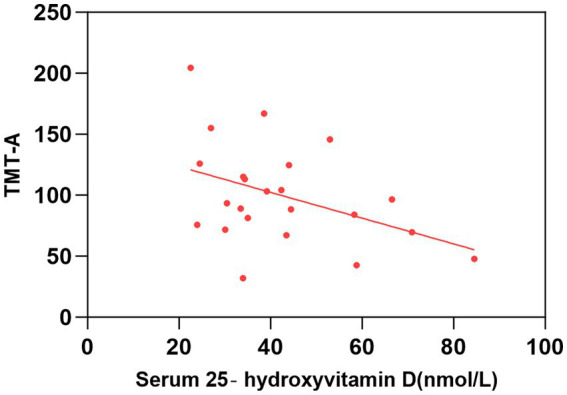
Correlation between serum 25-hydroxyvitamin D level and TMT-A. The simple linear regression analyse showed that serum 25-hydroxyvitamin D levels were negatively correlated with TMT-A scores (*P* < 0.05). TMT, Trail Making Test.

**Figure 3:**
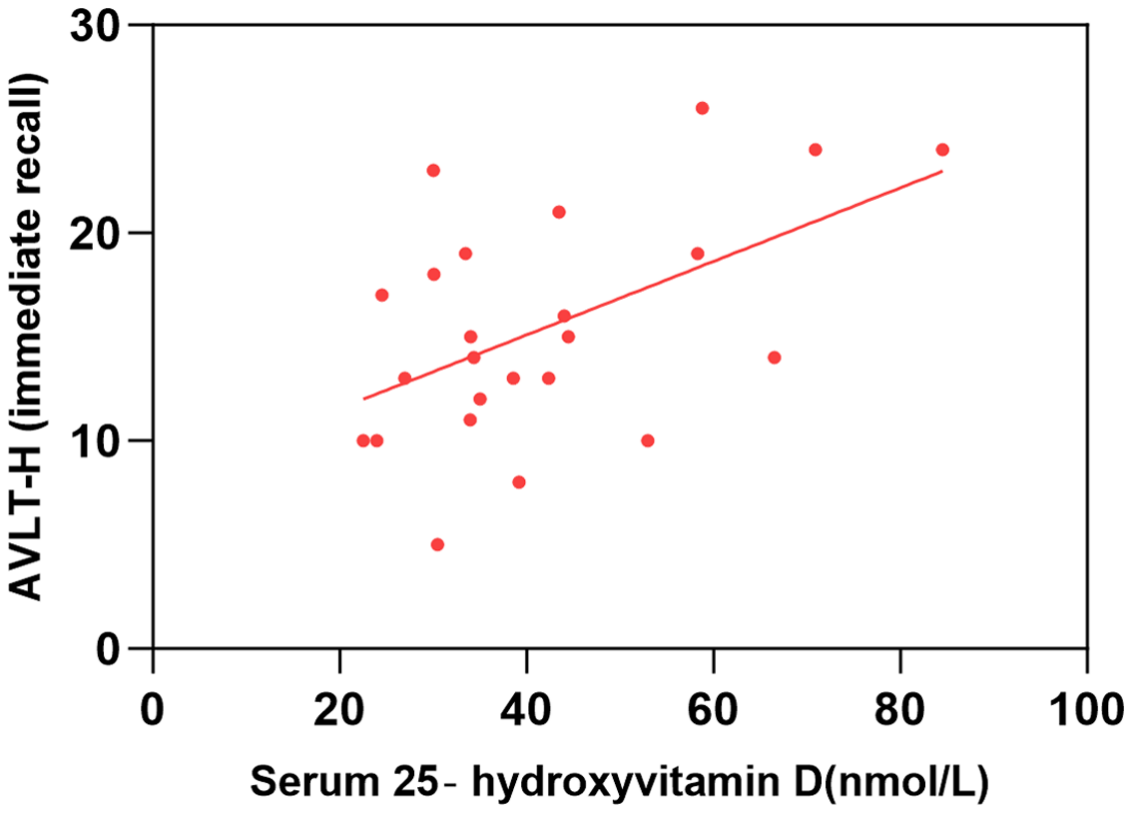
Correlation between serum 25-hydroxyvitamin D level and AVLT-H (immediate recall). The simple linear regression analyse showed that serum 25-hydroxyvitamin D levels were positively correlated with AVLT-H(immediate recall) scores (*P* < 0.05). AVLT-H, Auditory Verbal Learning Test-Huashan Version.

**Figure 4:**
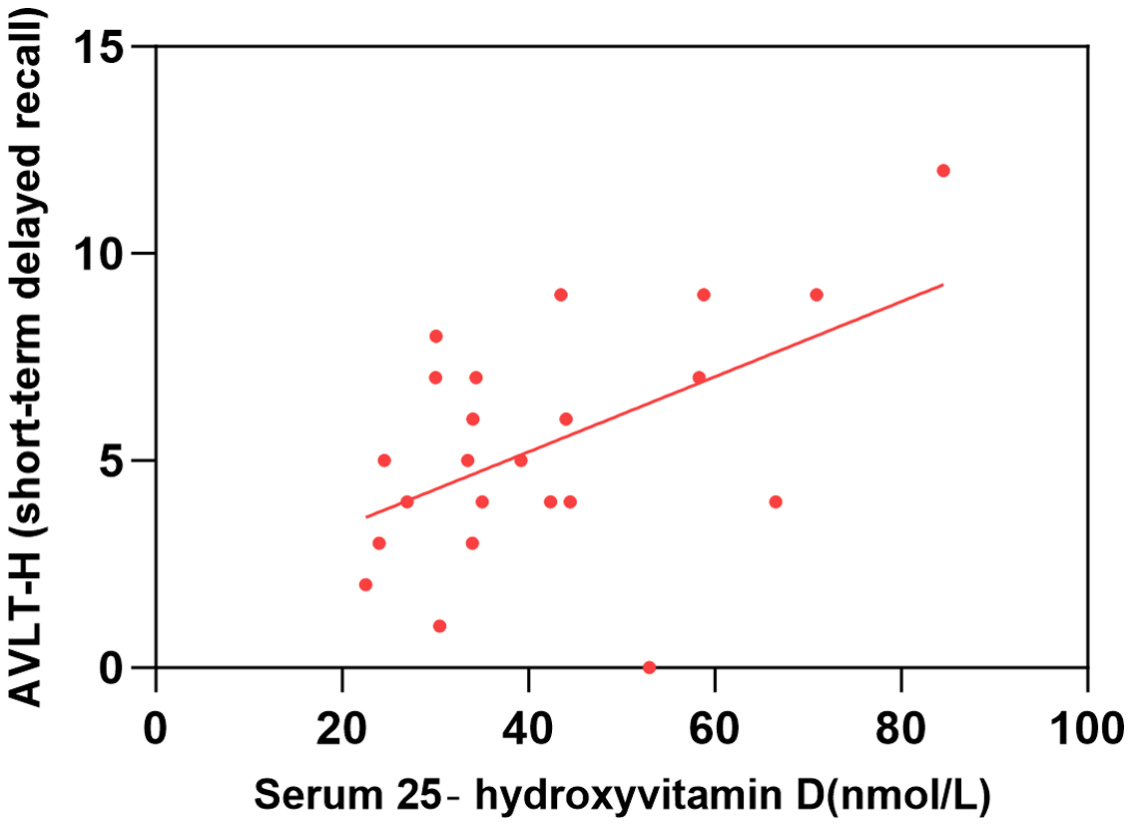
Correlation between serum 25-hydroxyvitamin D level and AVLT-H (short-term delayed recall). The simple linear regression analyse showed that serum 25-hydroxyvitamin D levels were positively correlated with AVLT-H(short-term delayed recall) scores (*P* < 0.05). AVLT-H, Auditory Verbal Learning Test-Huashan Version.

As shown in Table 2 and Figures 1–4, after adjusting for age, education level, UPDRSIII scores, and disease duration, the simple linear regression analyses between serum 25-hydroxyvitamin D levels and the scores of subtests that analyzed cognitive function showed that serum 25-hydroxyvitamin D levels were negatively correlated with TMT-A scores (*p* < 0.05) and positively correlated with SDMT and AVLT scores (*p* < 0.05).

**Table 2:** Simple linear regression analyses between serum 25-hydroxyvitamin D levels and cognitive function in PDND patients.

	Serum 25-hydroxyvitamin D levels in PDND patients		
	*Β*	*T*	*p*
DST (forward)	−0.003	−0.078	0.939
DST (backward)	−0.051	−0.477	0.639
SDMT (90 s)	0.367	3.726	0.002^**^
TMT-A	−1.066	−2.094	0.049^*^
TMT-B	−0.109	−0.655	0.514
SCWT	−0.021	0.101	0.921
AVLT-H (immediate recall)	0.177	2.845	0.009^**^
AVLT-H (short-term delayed recall)	0.093	2.791	0.011^*^
AVLT-H (long-term delayed recall)	0.379	2.052	0.054
AVLT-H (clue recall)	0.095	2.398	0.026^*^
AVLT-H (recognition)	0.052	2.284	0.035^*^
ROCF (copy)	0.015	0.129	0.899
ROCF [immediate recall (3 min)]	0.237	1.281	0.217
CDT	−0.062	−0.302	0.769
AFT (the first 15 s)	−0.008	−0.176	0.864
AFT (the last 45 s)	−0.049	−1.032	0.332
BNT (0)	−0.085	−0.466	0.647
BNT (1)	−0.035	−0.725	0.479
BNT (2)	0.040	0.200	0.844

DST, digit span test; SDMT, Symbol Digit Modalities Test; TMT, Trail Making Test; SCWT, Stroop Color-Word Test; AVLT-H, Auditory Verbal Learning Test-Huashan Version; ROCF, Rey–Osterrieth complex figure; CDT, Clock Drawing Test; AFT, animal fluency test; BNT, Boston Naming Test; PDND, Parkinson’s disease with non-dementia. **p < 0.01, *p < 0.05.

### 3.3 Serum 25-hydroxyvitamin D and GMV

**Figure 5:**
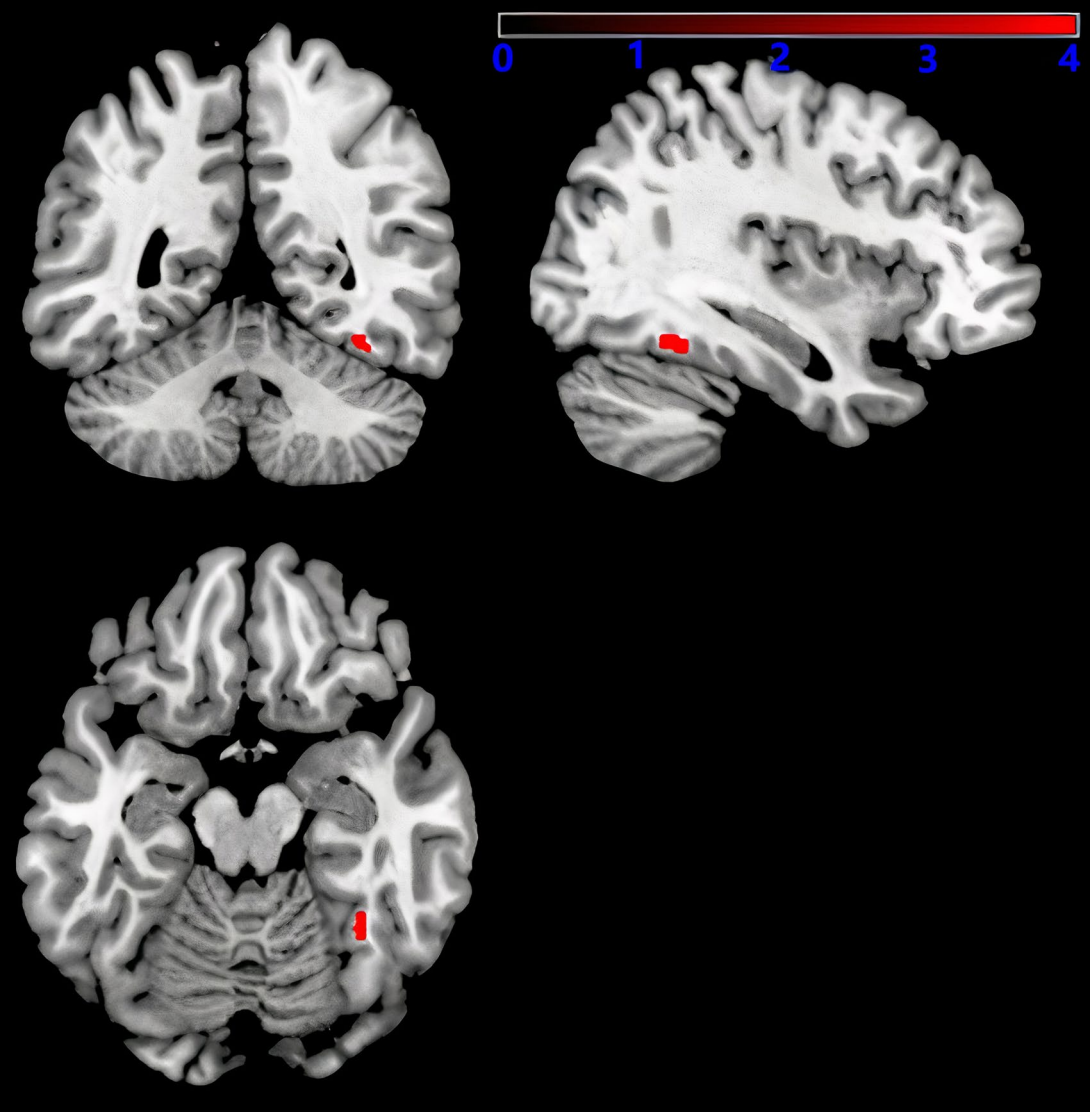
Serum 25-hydroxyvitamin D and GMV. Voxel-based morphometry results, showing the gray matter volume of the right fusiform gyrus, which positively correlated with serum 25-hydroxyvitamin D levels (red, uncorrected *P* < 0.001).

As shown in Figure 5, after adjusting for sex, age, education level, UPDRSIII score, disease duration, BMI, HAMD score, and intracranial volume, multiple regression analyses for whole brain GMV and serum 25-hydroxyvitamin D levels Multiple regression analyses revealed a positive correlation between the right fusiform gyrus GMV and serum 25-hydroxyvitamin D levels (uncorrected *p* < 0.001). The corresponding voxel number, Montreal Neurological Institute (MNI) coordinates, and *z*-values of the peak point in this region are shown in Table 3.

**Table 3:** Serum 25-hydroxyvitamin D levels are associated with some brain regions in PDND patients.

Active region	Side	*X*	*y*	*Z*	Activated voxel	*T*-value
Fusiform gyrus	Right	34.5	−51	−15	45	4.015

## 4 Discussion

Our analyses revealed a significant association between vitamin D, GMV and cognition in PDND patients. The serum 25-hydroxyvitamin D levels of the PDND group were significantly lower than those of the HC group. We also observed that serum 25-hydroxyvitamin D level was negatively correlated with worse performance on some cognitive tests in PDND patients and positively correlated with GMV in right fusiform gyrus.

Since Sato et al. (1997) first reported that PD patients had lower serum vitamin D levels in 1997, an increasing number of studies have found similar results. Evatt et al. (2008) found that PD patients had a higher incidence of vitamin D deficiency than healthy controls in their retrospective, cross-sectional study. Vitamin D is obtained from the diet and is synthesized in the skin, following exposure to solar ultraviolet B radiation. Kenborg et al. (2011) identified 3,819 men with a primary diagnosis of PD and selected 19,282 age-and sex-matched population controls and estimated odds ratios for the development of PD. Among groups divided according to whether they engaged in moderate, frequent, or maximal outdoor work, compared with exclusive indoor work, the result showed that men who work outdoors had a lower risk of developing PD (Kenborg et al., 2011). Wang et al. (2015) noted that gastrointestinal dysfunction, a frequent non-motor symptom of PD, could reduce vitamin absorption and account for lower level of vitamin D in PD patients. Cognitive impairment often associated with dysphagia, leading to reduced food intake (Fyfe, 2015). These findings were consistent with part of our study and might explain the reduction in vitamin D.

A large number of studies have proved that vitamin D has multiple functions in the nervous system. The functions of vitamin D are mediated through the vitamin D receptor (VDR). The VDR belongs to the superfamily of nuclear hormone receptors and serves as a ligand-induced transcription factor. VDR has been identified in the brains of rats and hamsters and is widely expressed in the adult temporal lobe (Gatto et al., 2016). Vitamin D appears to play a trophic role in the differentiation and maturation of neurons by regulating the rate of mitosis and the levels of neurotrophins, such as Nerve Growth Factor (NGF) and neurotrophin-3 (Dursun et al., 2011). Additionally, vitamin D promotes neuronal calcium homeostasis by downregulating the expression and density of calcium channels. It also exhibits anti-inflammatory effects in the brain, which aligns with the observed reduction in inflammatory brain injury following vitamin D repletion (Moore et al., 2005). Most studies suggested that vitamin D deficiency was positively correlated with cognitive status in the elderly, but some studies suggest that there’s no relationship (Moon, 2016). Iacopetta et al. (2020) have further suggested that vitamin D deficiency promotes the development of cognitive impairment in elderly patients with multiple complications. In recent years, there have been many studies focusing on the correlation between vitamin D deficiency and the cognitive status of PD. Most studies believe that there is a positive correlation between vitamin D deficiency and the cognitive status of the PD (Barichella et al., 2022; Lv et al., 2020; Peterson et al., 2013). In our study, we also observed that lower serum 25-hydroxyvitamin D level was associated with worse performance on some cognitive tests in PDND patients, which was consistent with most researches.

Many studies have described an association between vitamin D, GMV, and brain volume. Gray matter volume (GMV) is a marker of brain atrophy and a potential marker of cognitive function (Qin et al., 2022). The intake of vitamin B12, vitamin D, and zinc was positively associated with GMV in patients with normal cognitive function (Emre et al., 2007). Ali et al. (2020) found that the concentration of serum 25-hydroxyvitamin D was positively correlated with the GMV in the left calcarine sulcus among community-dwelling older adults. Shivakumar et al. (2015) discovered a significant positive correlation between vitamin D and the regional gray matter volume in the right hippocampus in schizophrenia. This observation supports a potential role of vitamin D deficiency in mediating hippocampal volume deficits, possibly via neurotrophic, neuroimmunomodulatory, and glutamatergic effect. In the AD subjects, the left parahippocampal, fusiform, and hippocampal regions showed a positive correlation with 25-hydroxyvitamin D. Meanwhile, low blood levels of 25-hydroxyvitamin D were found to be associated with a reduction in the volumes of the olfactory and hippocampal regions in elderly patients with cognitive decline (Lee et al., 2021). Furthermore, in AD patients, higher concentrations of 25-hydroxyvitamin D were found to be correlated with an enlargement in the white matter volume and the volumetric measure of the medial temporal lobe, including the amygdala and hippocampus (Hooshmand et al., 2014). VBM studies have demonstrated that patients with PD without dementia mainly present atrophy in frontal and temporal areas (Ibarretxe-Bilbao et al., 2009). Although the brain regions involved in different diseases are not entirely identical, a significant number of them are concentrated in the occipital and temporal lobes. In our study, we found the precise brain region, that GMV in right fusiform gyrus was positively associated with serum 25-hydroxyvitamin D level in PDND.

The fusiform gyrus is involved in a range of visual cognitive functions, such as face recognition. The fusiform gyrus can be divided into three subregions: medial fusiform gyrus (FGm), lateral fusiform gyrus (FGl), and anterior fusiform gyrus (FGa). FGm may be involved in low-level visual processing and bilateral FGm is positively and functionally connected with the auditory network (Caspers et al., 2014; Hauw et al., 2024). FGl has also been identified to play a key role in various visual cognition (Wang et al., 2013). Based on whole-brain functional connectivity maps, studies have also found that the FGa was functionally correlated with the middle temporal gyrus and the middle occipital area, which predicts its role in audiovisual functions (Feng et al., 2018). Our study found that serum 25-hydroxyvitamin D in PDND patients was significantly positively related to auditory word learning and spatial cognition, and we also found that the lower serum 25-hydroxyvitamin D levels, the less GMV in the right fusiform. Considering the role of the fusiform gyrus in cognition, we hypothesized that the lower serum 25-hydroxyvitamin D level in patients with PDND might affect auditory word learning and spatial cognition ability by reducing the gray matter volume of the right fusiform gyrus, thereby leading to deterioration of semantic understanding and memory function.

In summary, we found atrophy of the right fusiform gyrus with lower serum 25-hydroxyvitamin D level in PDND patients, and serum 25-hydroxyvitamin D level was closely positive associated with cognition. This finding highlights that specific brain areas are altered with low vitamin D level in PD patients, and help understanding the effects of vitamin D on cognition in PDND patients. In the future, large-scale cohort studies should be conducted to further solidify the results of this research. We could predict the cognitive condition of PD patients by detecting the concentration of vitamin D. More randomized double-blind studies can be used to further clarify whether vitamin D supplementation can effectively improve the volume of relevant brain regions and cognitive function of patients with PDND.

Our study has several limitations. Firstly, we did not find any association between the whole brain and other cognitive domains, which may be due to the small sample. Secondly, based on the patient’s ability to complete the whole scale assessment and prolonged magnetic resonance examination, we chose PDND patients as the subject of our study. To some extent, this may cause selection bias. Thirdly, small sample sizes render it hard to represent the actual condition of the population precisely and can result in decreased stability of effect size, less accurate estimation, and a chance of overlooking minor effects. Additionally, small sample sizes can reduce statistical power, thus heightening the risk of false-negative results. Fourthly, patients who took oral vitamin D supplements were excluded from our study, but we did not calculate the potential effects of dietary habits and sun exposure on vitamin D levels, factors that could confound the results.

## Data Availability

The raw data supporting the conclusions of this article will be made available by the authors, without undue reservation.
